# Chronic Viral Hepatitis in Elite Athletes: Approaches to Risk Assessment, Prevention and Management

**DOI:** 10.1186/s40798-022-00517-9

**Published:** 2022-10-04

**Authors:** Lung-Yi Mak, Ian Beasley, Patrick T. F. Kennedy

**Affiliations:** 1 Department of Immunobiology, Barts Liver Centre, The Blizard Institute, Barts and The London School of Medicine and Dentistry, Queen Mary University of London, London, UK; 2 Centre for Sports and Exercise Medicine, Queen Mary College, London, UK; 3 Department of Medicine, School of Clinical Medicine, Queen Mary Hospital, The University of Hong Kong, Hong Kong, China; 4 State Key Laboratory of Liver Research, The University of Hong Kong, Hong Kong, China

**Keywords:** HBV, HCV, HDV, Blood-borne virus, Collision sports, Contact sports, Sports medicine, Vaccination, Liver cancer, Cirrhosis

## Abstract

Elite athletes who participate in contact sports are at risk of bleeding injuries, leading to transmission of blood-borne viruses including hepatitis type B, C and D (HBV, HCV and HDV) capable of causing chronic liver disease, liver failure and liver cancer. In view of the significant advances in the viral hepatitis field over the past decade, more structured approaches should be in place to screen for and manage viral hepatitis in elite athletes. HBV status should be assessed in all elite athletes, and those infected should receive nucleos(t)ide analogues for viral suppression, while uninfected individuals should receive HBV vaccination. The all-oral direct acting antivirals for HCV are highly effective and safe, thus the remaining challenge with hepatitis C is case identification and linkage to care. HDV is only found in HBV-infected individuals, which is characterized by rapid disease progression and higher rates of cirrhosis and liver cancer in infected subjects. Pegylated interferon was the mainstay of treatment for HDV infection until bulevirtide, a viral entry inhibitor, was recently approved by the European Union (EMA) and FDA in America, while multiple novel therapies are already in clinical trials as part of the HBV cure program. Overall, awareness of chronic viral hepatitis in athletes should be improved. Prevention remains the cornerstone of the management of viral hepatitis in sport coupled with rigorous disease assessment in infected individuals, and antiviral therapy where indicated.

## Key Points

- Blood-borne hepatotropic viruses (hepatitis type B, C and D [HBV, HCV and HDV]) are capable of transmission in athletes of collision/contact sports due to bleeding injuries.
- Potentially serious liver complications from HBV, HCV and HDV can be minimized by effective preventive and therapeutic strategies.
- Elite athletes with HBV should be virally suppressed, and uninfected subjects should be vaccinated; those participating in collision/contact sports should aim for low viral load and confirmation of vaccine response, respectively.
- Remarkable progress has been made in the HCV field and the virus can be cured with 2–3 months of effective, safe, all-oral direct acting antiviral therapy.
- Awareness of HDV, which causes rapid disease progression among HBV infected individuals, must improve; a new therapy has recently been approved.

## Background

Hepatitis viruses type A–E are capable of infecting human livers and leading to various clinical sequelae. While both hepatitis A and E are ‘food-borne’ viruses and mainly cause acute self-limiting illnesses, hepatitis type B, C and D are ‘blood-borne’ viruses and result in chronic liver disease (Table 1). Globally, chronic viral hepatitis caused by hepatitis B virus (HBV) and hepatitis C virus (HCV) affects 325 million people, while hepatitis D virus (HDV) affects an estimated 12 million people, approximately 4.5% of those chronically infected with HBV [1]. A total of 1.34 million people died from viral hepatitis-related chronic liver disease or hepatocellular carcinoma (HCC) in 2015, which was comparable to deaths caused by tuberculosis and higher than those caused by human immunodeficiency virus (HIV) [2]. Transmission of hepatitis viruses, such as HBV, has been documented by non-conventional routes among athletes participating in contact sports like wrestling [3–5] and football [6] due to close contact of the subjects and bleeding from combat activities and collisions occurring in any contact sport [7, 8]. The risks of transmission of hepatitis viruses among elite athletes are hard to quantify owing to the scarcity of data in the literature. There are no standardized policies to address the growing need to prevent transmission of viral hepatitis like HBV among elite athletes, especially with globalization of sport and the growing numbers of elite athletes from HBV endemic countries. Significant progress has been made in our understanding of viral liver disease and major breakthroughs have been made in the management of viral hepatitis in the last decade. This review is aimed to review the current literature, discuss the significance of this progress in the field and the approach to management of chronic viral hepatitis caused by HBV, HCV and HDV among elite athletes, such that they can achieve their career goals and overcome the obstacles associated with a diagnosis of viral liver disease. This guidance may be potentially useful for all athletes and is especially applicable to elite athletes who could be excluded from their profession if they are living with chronic viral hepatitis, because of a lack of awareness and understanding of viral liver disease in sport.

**Table 1:** Overview of hepatitis viruses capable of causing human liver disease

	Hepatitis A virus	Hepatitis B virus	Hepatitis C virus	Hepatitis D virus	Hepatitis E virus
Nature	RNA virus	DNA virus	RNA virus	RNA virus	RNA virus
Main route of transmission	Faecal-oral route	Percutaneous route	Percutaneous route	Percutaneous route	Faecal-oral route
Common clinical presentations	Asymptomatic	Asymptomatic	Asymptomatic	New onset/ persistent deranged liver function in HBV + subjects	Asymptomatic
					Self-limiting GI upset
	Self-limiting GI upset	Incidental finding of deranged liver function			Acute icteric illness
	Acute icteric illness				
Clinical importance	Acute liver failure	Acute liver failure	Acute icteric illness (for recently acquired HCV)	Rapid progression of liver disease in HBV + subjects	Acute liver failure
		Acute on chronic liver failure			Fulminant hepatitis in pregnant women
		Cirrhosis			Chronic allograft dysfunction for post-liver transplant recipients
		HCC	Cirrhosis		
			HCC		
Chronicity	No	Yes	Yes	Yes	No except for immunocompromised hosts
Treatment	Supportive treatment	Available	Available (curative)	Available	Supportive treatment
Vaccine available	Yes	Yes	No	No	No (except one vaccine licensed for use in China in year 2011; currently being evaluated in clinical trial in USA)

*HBV* hepatitis B virus; *HCC* hepatocellular carcinoma; *HCV* hepatitis C virus; *GI* gastro-intestinal; *USA* United States of America

### Hepatitis B Virus

#### Epidemiology and Clinical Implications

In the most updated report published in 2021 [9], the World Health Organization (WHO) estimates that 296 million (3.8% of the global population) were living with HBV globally. The African continent (82 million; 7.5%) and Western Pacific (116 million; 5.9%) region accounted for 67% of all infections, followed by South-east Asia (60 million; 3.0%), Eastern Mediterranean (18 million; 2.5%), Europe (14 million; 1.5%), and the Americas (5 million; 0.5%) [9].

HBV is a partially double-stranded DNA virus and can lead to both acute and chronic hepatitis B (CHB) infection in humans (Table 1). Age of exposure is the main factor that determines the development of chronic infection. Exposure during infancy or early childhood leads to chronic infection in the majority of cases, whereas exposure to the virus later in life is mostly self-limiting, with < 5% developing chronic infection [10, 11], while a minority of cases can progress to acute liver failure (1%) [12] and even death.

CHB is one of the leading causes of HCC and cirrhosis. It is estimated that 25% of untreated individuals with chronic infection will die of these complications [13]. Of note, HCC can also develop in non-cirrhotic HBV-infected livers. CHB infection is diagnosed when serum hepatitis B surface antigen (HBsAg) is detected in the blood on two occasions 6 months apart. It is classified into different disease phases by serological and biochemical markers, namely hepatitis B e antigen (HBeAg) positivity/negativity, HBV deoxy-ribonucleic acid (HBV DNA) level, and serum alanine aminotransferase (ALT)—a marker reflecting liver necro-inflammation. HBV DNA levels are usually higher in HBeAg-positive versus HBeAg-negative disease phases. Nevertheless, HBV DNA levels fluctuate as well as the corresponding ALT regardless of HBeAg positivity.

#### Diagnosis

In individuals exposed to HBV during adulthood, the most likely clinical outcome is a self-limiting illness, and thereby a temporary (< 6 months) detection of HBsAg in the blood. Many cases are asymptomatic, although some can present with jaundice or rarely liver failure. During acute infection, antibody to hepatitis B core antigen (anti-HBc) will be detected alongside other serological markers including HBsAg, HBV DNA, with or without HBeAg (Table 2). However, potential pitfalls when using HBsAg should be noted, e.g. a negative result in the very early phase of infection, i.e., ‘window period’—defined as the period when there is active viral replication but the subject is still seronegative for HBsAg—has been well characterized in blood donation units [14]. If clinical suspicion is high, typically when an adult without prior liver disease presents with acute icteric illness, elevated serum ALT, or acute liver failure, serological markers should be repeated or more serological assays should be performed, such as the measurement of HBV DNA. Another scenario when HBsAg may be falsely negative is in the presence of HBsAg mutations, which are typically induced by the immune selection pressure imposed from active (see 'Vaccination for HBsAg Negative Sportspersons' below) [15] or passive immunization (HBV monoclonal antibodies) [16], when the induced conformational changes in the mutated HBsAg lead to undetectability by the currently available assays. Other HBV viral markers should be detectable in the serum (including HBV DNA, HBeAg, anti-HBe antibody and anti-HBcore; see Table 2) in these subjects.

**Table 2:** Interpretation of hepatitis B virus blood tests

HBsAg	Anti-HBc	Anti-HBs	HBeAg	HBV DNA	Interpretation
+	+	−	+ or −	+	Recently acquired HBV
					Chronic hepatitis B infection (if persists for ≥ 6 months)
−	+	−	−	+	Occult hepatitis B infection from chronic carriers who lose the HBsAg
					Occult hepatitis B infection from chronic carriers with surface antigen mutation
					‘Window period’ of recently acquired HBV
−	+	−	−	−	Resolved acute infection
−	−	+	−	−	Immunity from vaccination
−	+	+	−	−	Immunity from natural infection
−	−	−	−	−	No prior exposure to HBV
					No protective antibody response following vaccination
−	−	−	−	+	‘Window period’ of recently acquired HBV

*Anti-HBc* antibody to hepatitis B core antigen; *anti-HBs* antibody to hepatitis B surface antigen; *DNA* deoxy-ribonucleic acid; *HBeAg* hepatitis e antigen; *HBV* hepatitis B virus

Even after clinical resolution of acute HBV infection, the virus will persist in the liver in the form of covalently closed circular DNA (cccDNA) and can lead to occult HBV infection. The clinical impact is negligible for most cases of occult HBV infection following resolution of acute HBV. It has been characterized that patients with occult hepatitis B infection have a low viremic status in the range of a few hundred copies per millilitres (copies/mL) in the blood [17]. However, some patients with occult HBV infection can harbour higher viral loads. In a cohort of Turkish Olympic wrestlers who were all HBsAg-negative, the HBV DNA was as high as 7900 copies/mL in the blood (and 7500 copies/mL in the sweat) of a wrestler with positive serum anti-HBc [18]. More importantly, the study found that HBV viral load in the blood correlated with the viral load in sweat (*r* = 0.52) [18]. Indeed, bodily fluids can contain actively replicating viruses and are capable of causing infection as demonstrated in an experiment where the tear of an HBV-infected child caused HBV infection in two human hepatocyte-transplanted mice via hematogenous contact [19]. This is further illustrated by a case report of acute HBV in 3 wrestlers in the same club, which showed that horizontal transmission of HBV is possible even without sexual or bleeding contact [5]. Therefore, bodily fluids other than blood or semen, such as sweat, are theoretically capable of transmission of HBV to close contacts.

Taken together, HBV infection can lead to serious clinical sequelae and is a transmittable disease among athletes.

#### Determinants for Risk of HBV Transmission

**Fig. 1:**
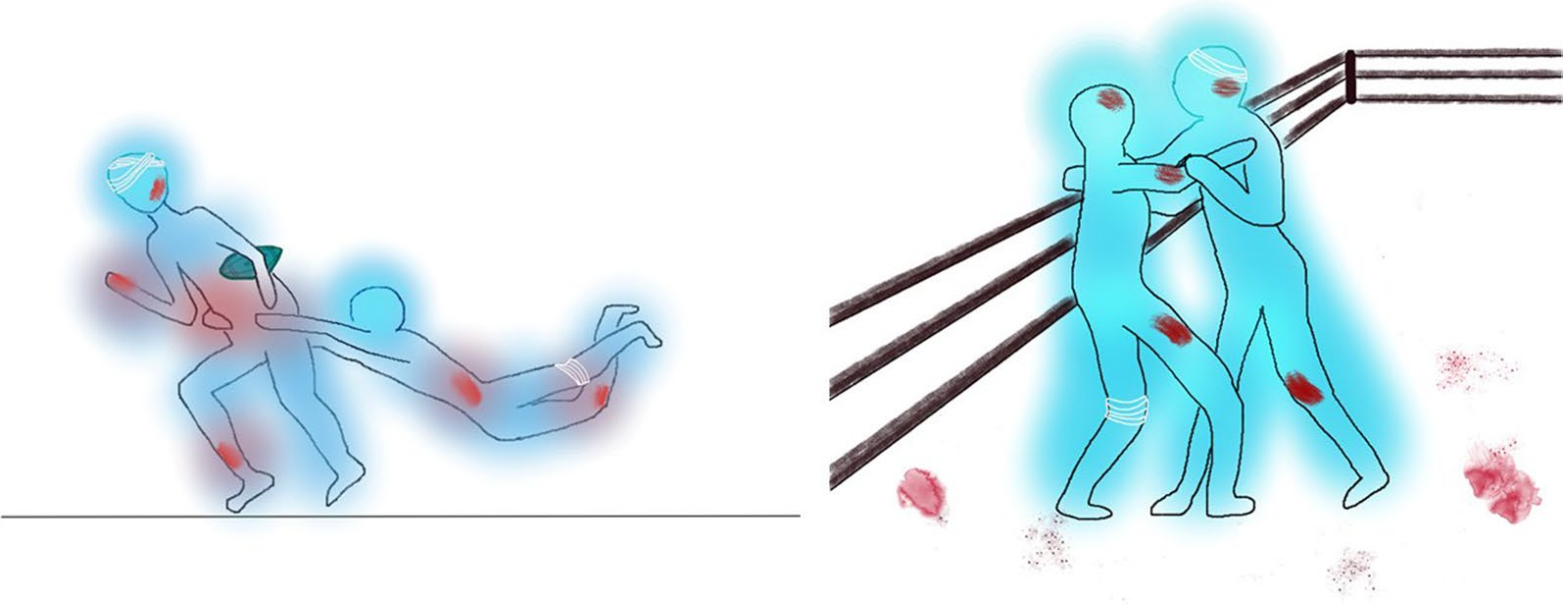
Cartoon showing the risk of bleeding (represented in red colour patches and white bandages) in athletes participating in full contact/ collision sports. The left panel represents rugby, and the right panel represents wrestling

**Fig. 2:**
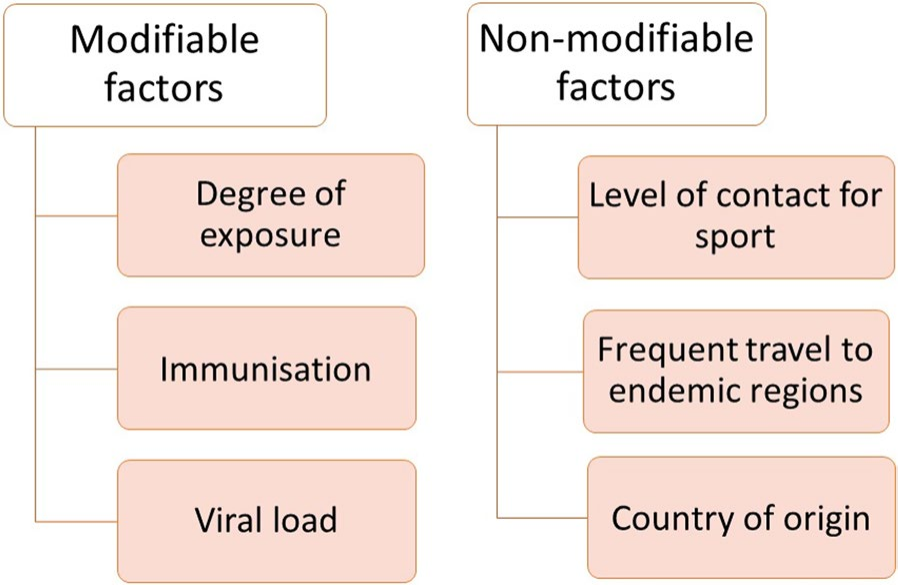
Factors determining the risk of HBV transmission among elite athletes

The theoretical risk of HBV transmission among athletes was estimated to be between one transmission in every 850,000 to 4,250,000 games of contact sport and one transmission in every 10,000 to 50,000 games based on the same methods of risk calculation used for HIV [20]. In principle, the risk of HBV transmission is proportional to the viral load in the index case. In previous reports of HBV transmission among wrestlers, the viral load in those with acute hepatitis B virus infection ranged from 4.3 to 7.2 logs [5], in mostly HBeAg-positive subjects. Moreover, the vulnerability of individuals to the HBV-infected bodily fluid depends on the degree of exposure, i.e., presence of wounds and mucosal contact, and whether the individuals are immunised against HBV (see 'Vaccination for HBsAg Negative Sportspersons' below). The degree of exposure is closely related to the level of contact for different kinds of sports in addition to the chance of bleeding and sustaining open wounds. It is difficult to estimate the risk for each specific type of sport, and by classifying them into broad categories according to the level of contact will aid risk-stratification (Table 3). Full contact/collision sports are those where significant physical impact force is incurred on players, either deliberately or incidentally, and is allowed or within the rules of the game (Fig. 1). Semi-contact sports involve striking and physical contact between players, where the contact is not really part of the game, but is generally accepted as is the case with football. Low or non-contact sports are those in which players are not expected to touch other people and thus, contact is not part of the game. Full contact sports are deemed to bear the highest risk of HBV transmission—indeed prior reports of HBV transmission among athletes were mostly from this group, whereas low or non-contact sports are deemed to bear the lowest risk. Lastly, risk of an athlete acquiring HBV will also depend on the opponent’s country of origin, especially those from highly endemic areas, because prevalence rates of HBV are variable across different geographical locations. Highly endemic countries are defined when local prevalence rate is > 8%, whereas low endemic countries are defined when local prevalence rate is < 2%; and countries are defined as intermediately endemic with prevalence rates of 2–8% [21]. Occasionally, an athlete may need to be hospitalized in a country that is endemic for HBV, and the healthcare-associated risks of exposure to HBV should also be considered. Figure 2 outlines the above factors determining the risk of HBV transmission among elite athletes.

**Table 3:** Level of contact for different sports and examples

	Full contact/ collision sports	Semi-contact sports	Low/non-contact sports
Definition	Any sport for which significant physical impact force on players, either deliberate or incidental, is allowed or within the rules of the game	Combat sport involving striking and physical contact between the players. The contact is not really part of the game but is accepted	The player is not expected to touch other people and such contact is not part of the game
Examples	Football Rugby Wrestling Mixed martial arts* Hockey Lacrosse Water polo Boxing* Taekwondo	Basketball Volleyball Handball	Running Rowing Sailing Swimming Tennis Badminton Table tennis Weight lifting Field events (e.g., javelin, discus) Golf Gymnastics Snooker Diving Bicycle race Archery

^*^Prohibited from sport if diagnosed with hepatitis B infection

#### Prevention of HBV Transmission

**Fig. 3:**
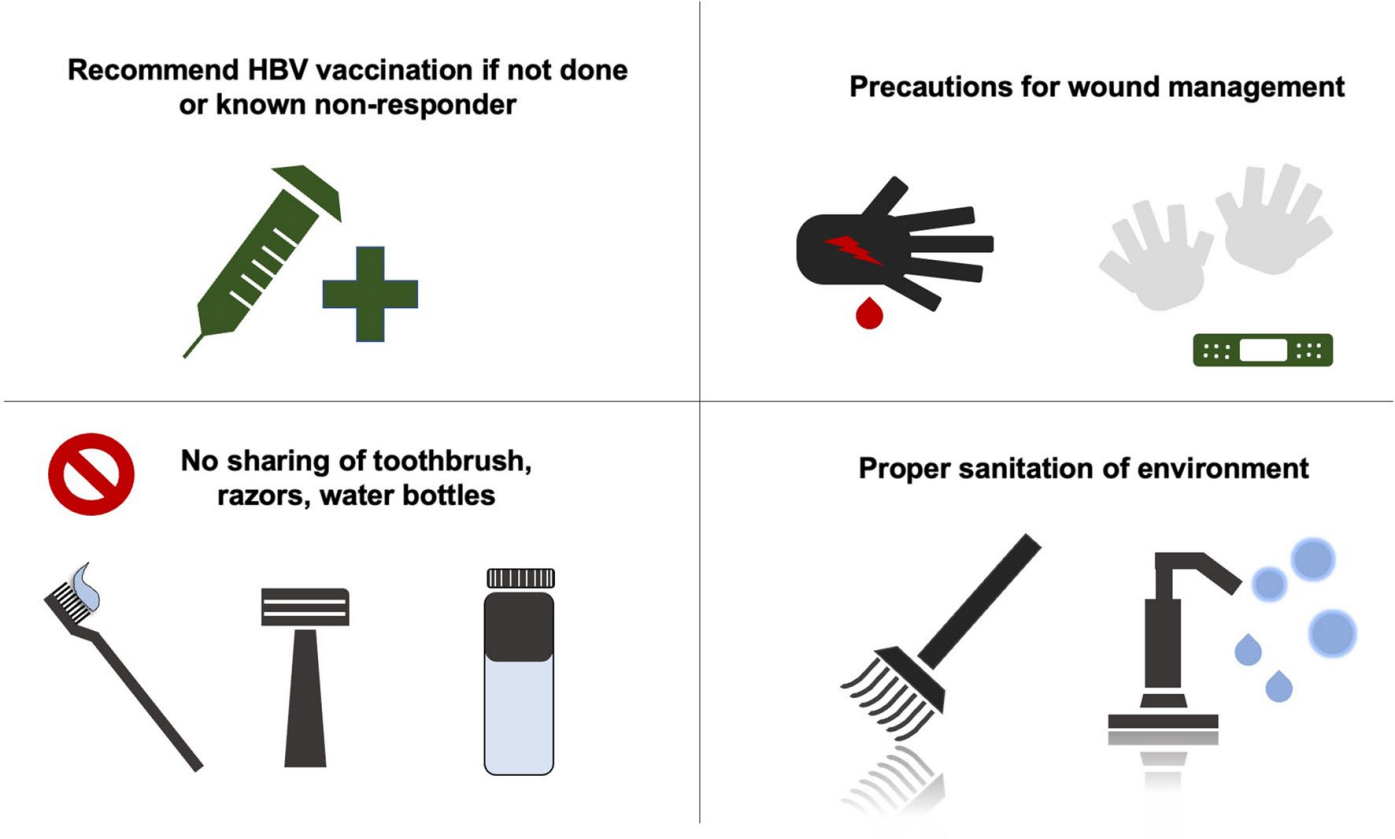
Recommended universal actions for prevention of spread of HBV among athletes

**Fig. 4:**
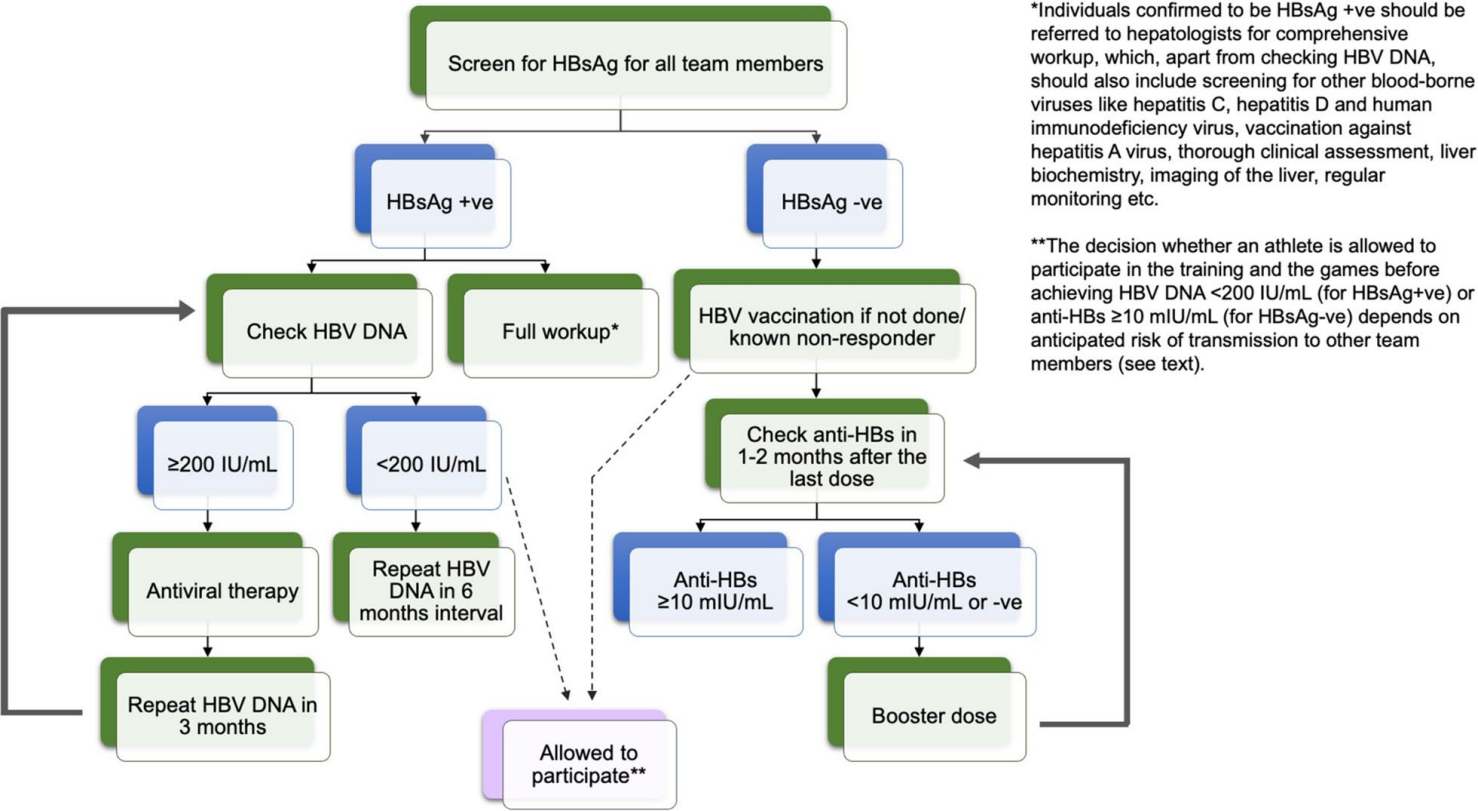
Recommended actions for screening and treatment of HBV among elite athletes. *Anti-HBs: antibody to hepatitis B surface antigen, HBsAg: hepatitis B surface antigen, HBV DNA: hepatitis B virus deoxy-ribonucleic acid*

We propose the following measures for prevention of spread of HBV among elite athletes. Firstly, for every team member (including the playing or coaching staff, referees, judges, certified athletic trainers and team physicians), HBV vaccination is recommended (see 'Vaccination for HBsAg Negative Sportspersons' below). Secondly, strict precautions for wound management should be followed, including the application of universal precautions by means of protective gloves and physical barriers to open wounds. Thirdly, it should be advised that personal items like razors, toothbrushes and water bottles (which may harbour blood-contaminated saliva or sweat and thus) should not be shared. Needle sharing for medications or illicit drug usage must be prohibited. Finally, the common areas of the environment such as the changing rooms and desks/ benches should be appropriately sanitized. HBV has been reported to be inactivated by highly-concentrated disinfectant chemicals such as 70% isopropyl alcohol [22] and sodium hypochlorite [23] in non-human studies. Although no definitive recommendations can be made on the type of disinfectant, it is always prudent to maintain a hygienic environment to reduce risk of HBV transmission from inadvertent contact with infected bodily fluid (Fig. 3). For elite athletes who have never been tested for HBsAg, their blood should be screened for HBsAg to guide whether they should be vaccinated (if HBsAg-negative) or treated (if HBsAg-positive) (Fig. 4).

#### Management of HBsAg-Positive Sportspersons

Being HBsAg-positive requires further evaluation and potentially antiviral therapy. However, it is noted that certain full contact sports prohibit any HBsAg-positive players from participating in the game, such as boxing and mixed martial arts [24]. Taking reference to health care professionals (HCPs) as an example, exposure-prone procedures (EPP) can still be performed by HCPs as long as viral load is suppressed to a certain threshold. According to the United Kingdom Advisory Panel (UKAP) for Healthcare Workers Infected with Blood-borne Viruses, HCPs with HBV infection are allowed to perform EPP once the serum HBV DNA is below 200 IU/mL either spontaneously or by antiviral therapy [25], whereas the cut-off is slightly higher (1000 IU/mL) according to the Centers for Disease Control and Prevention (CDC) recommendations [26]. A low HBV DNA threshold was chosen based on the previous reports of HBV transmission from HCPs to patients that the lowest measured viral load was 2.5 × 10^5^ copies per mL in a HBeAg-negative surgeon. Virtually all HBV transmission associated with EPPs of HCPs occurred at HBV DNA levels > 2 × 10^4^ IU/mL [27, 28]. In the case of elite athletes, there is no evidence regarding the best cut-off level below which HBV-infected individuals are deemed safe to participate in sport while protecting other team members and opponents. More research should be conducted to assess the issue of setting a threshold of HBV viral load to prevent athlete-to-athlete transmission. Before more data become available, however, we advocate for a stringent criterion as in the case of HCPs performing EPP, i.e., 200 IU/mL, to minimize the risk of HBV transmission among elite athletes.

Checking the HBV DNA level is important to guide whether antiviral treatment should be started. Currently approved therapies for chronic HBV infection include both pegylated interferon (PEG-IFN) and oral nucleos(t)ide analogues (NUCs), and the latter is the preferred option for elite athletes owing to its high potency in viral suppression and low-risk of adverse events. Both drug groups are not on the Prohibited List of substances according to the World Anti-Doping Agency (WADA) [29]. In addition, the current first-line NUCs are generally very safe, taken as a daily tablet, and do not require intensive therapeutic monitoring. Suppression of HBV DNA to the desired range usually takes a few months of NUC therapy, and the rate of HBV DNA undetectability at 12 months ranges from 64 to 76% for HBeAg-positive subjects and 90–94% for HBeAg-negative subjects [30, 31]. Moreover, an HBsAg-positive elite athlete should be evaluated by a hepatologist to include comprehensive clinical assessment, regular blood monitoring of the liver enzymes, abdominal imaging to detect liver-related complications, vaccinating their household contacts and partners, and to control other risk factors for liver disease progression (e.g., alcohol and diet), in addition to screening for HCV and HDV (see below) as well as HIV. Furthermore, individuals with CHB should be vaccinated against hepatitis A virus if not already immune [30, 32]. Due to the nature of viral persistence, antiviral treatment is aimed at controlling the viral replication, but not clearance of virus from the body. Numerous novel therapies are currently being developed, aiming to enhance viral control and to improve the long-term outcomes of chronic HBV carriers without the need of indefinite or lifelong therapy. Comprehensive reviews of new drugs for chronic HBV are available elsewhere [33, 34] and will not be discussed in this article.

#### Vaccination for HBsAg-Negative Sportspersons

Universal HBV vaccination among infants, children and adolescents is advocated by the CDC Advisory Committee on Immunization Practices (ACIP). In April 2022, the ACIP updated the recommendations that all adults aged 19–59 should receive HBV vaccines without the need of risk factor screening [35], and the vast majority of elite athletes should fall into this age group. In the UK, all babies born on or after 1 August 2017 are given 3 doses of hepatitis B-containing vaccine as part of the National Health System routine vaccination schedule. These doses are given at 8, 12 and 16 weeks of age. Babies at high risk of developing hepatitis B infection from infected mothers are given extra doses of the hepatitis B vaccine at birth, 4 weeks and 1 year of age. In the United States, since 1991, the recommendation is that all medically stable infants born with a birth weight of ≥ 2000 g should receive the first dose of HBV vaccine within 24 h of birth, followed by two additional doses at 1 month and 6 months of age. A similar 3-dose vaccine series is recommended for children and adults. These are single HBsAg antigen vaccines including *Engerix-B*®, *Recombivax HB*®, and *Twinrix*® (in combination with HAV) and *PreHevbrio*® (Sci-B-Vac) which is a triple antigen vaccine (see below). *Heplisav-B* is a single antigen vaccine in combination with a toll like receptor 9 agonist (CpG-1018) that needs just two doses at 0 and 1 month for adults.

To define vaccine response, also known as anamnestic response, serum antibody to HBsAg (anti-HBs) should be ≥ 10 international units per litre (IU/L) or milli-international units per milli-litre (mIU/mL). As many currently available recombinant yeast-derived HBsAg-containing vaccines are derived from HBV genotype A2, some in vitro studies have shown insufficient neutralization of non-genotype A HBV at low anti-HBs titres [36]. Nevertheless, the current HBV-A2 vaccines are highly effective in preventing infections and clinical disease caused by all known HBV genotypes [37, 38]. Subjects who received the HBV vaccine have long-lasting immunological memory. After primary vaccination with a recombinant 3-dose vaccine in 146 infants, 47.9% had protective levels of anti-HBs after 10 years [39]. Although antibody levels may drop after vaccination [40] [41], this varies depending on the endemicity of HBV in that region. A Canadian study showed that anti-HBs levels persisted in 85% of subjects at 10 years [42], whereas 64% of Italian children maintained protective anti-HBs levels 10 years after vaccination [43]. Notably, despite low or declining anti-HBs levels, HBV infection is uncommon in persons known to have responded to the primary vaccine series. Immunological memory capable of preventing chronic HBV infection or symptomatic infection (e.g., early anti-HBs seroconversion, in vitro B cell stimulation of functional memory B lymphocytes) [44–46] persists even after anti-HBs decline to undetectable concentrations. Therefore, routine booster dose of vaccine is not recommended in the general population [47], but is recommended if anti-HBs level is below 10 mIU/mL among at-risk elite athletes.

Following screening / vaccination for HBV, the decision whether an elite athlete is allowed to participate in or return to training/competition depends on two factors. Firstly, whether the target viral load of HBV DNA < 200 IU/mL (for HBsAg-positive persons) or protective antibody level of anti-HBs > 10 mIU/mL (for HBsAg-negative persons) has been achieved. Secondly, the anticipated risk of transmission of the virus when the target has not been achieved, which is mainly determined by the level of contact for the sport—full contact/collision sports bear higher risk compared to semi-contact sports with virtually no risk in low or non-contact sports. Although limited data are available, HBV-positive elite athletes who participate in full contact/collision sports are recommended to refrain from training/ competition until HBV DNA < 200 IU/mL is achieved. In the event of a persistent vaccine non-responder, one should consider repeating HBsAg and/or checking HBV DNA to identify occult HBV carriers or a possible “window period” of recently acquired HBV infection. After excluding HBV infection, vaccine non-responders should be revaccinated with additional strategies that are proven to be efficacious to improve immune response. These include doubling the dose of vaccine [48], administering the vaccine via intradermal route (rather than intramuscular route) [49], or choosing an alternative vaccine that contains more immunogenic antigens like the pre-S1 and pre-S2 proteins in addition to the small HBsAg (e.g., *PreHevbrio*®) [50].

Overall, HBV vaccination is safe and has demonstrated long-term effectiveness for protection against HBV infection. It should be recommended for all elite athletes, and sero-protection should be confirmed in high-risk individuals, which would include those participating in full contact/ collision or semi-contact sports, and in the presence of other risk factors as discussed above. An accelerated schedule for recombinant HBV vaccine *Engerix®* at 0-7-21 days has been licensed for use in circumstances where adults are at immediate risk and a more rapid induction of protection is required [51]—a consideration for elite athletes to minimize interruption to training/ competitions while awaiting the completion of vaccine. An extra 4th dose is recommended at 12 months after the initial 3-dose vaccine to provide long-term protection [52].

### Hepatitis D Virus

#### Epidemiology & Clinical Implications

HDV is the smallest human virus with a diameter of 35–36 nm. As HDV requires HBV to help with entering hepatocytes [53] and spread (but not with its replication cycle), only subjects with HBV will be affected and the epidemiology largely follows that of HBV. It is estimated that 12 million people are infected with HDV globally, but due to low awareness, this figure maybe under-reported. The most prevalent areas include Mongolia, Uzbekistan, Central Asia, the Middle East, Central Africa and parts of South America [1]. The prevalence of HDV is estimated to be 4.5% among HBV + subjects, and more commonly seen in HBV-related cirrhotics (18%) and HBV-HCC (20%) [1]. When exposed to both viruses simultaneously, i.e., co-infection, most immunocompetent adults can achieve spontaneous resolution, with similar rates of progression to chronicity as observed in adults acquiring HBV [54] (< 5%; see 'Hepatitis B Virus' section), but the clinical course of acute HBV + HDV bears much higher risk of acute liver failure (20%) than HBV mono-infection [55]. For an HBV-infected subject, superinfection with HDV will lead to persistent HBV/HDV co-infection and accelerated disease progression [56]. The main bulk of disease is among chronically HBV-infected individuals, who are at risk of faster progression to cirrhosis and HCC. It is estimated that 50–70% progress to cirrhosis within 5–10 years of diagnosis [57, 58], with a rapid time to decompensation [21 months] [59] and > twofold higher risk of HCC compared to HBV mono-infected individuals [60–63].

#### Diagnosis

The European Association for the Study of Liver (EASL) guidelines recommend screening of HDV infection in all patients with CHB infection [30], while the American Association for the Study of Liver Diseases (AASLD) guidelines recommend screening for HDV among high-risk CHB patients [32]. The clinical suspicion for HDV superinfection is especially raised when an HBV-infected individual presents with new onset or persistently deranged liver enzymes despite NUC for HBV, or a rapidly progressive course of liver disease (Table 1). In addition, acute HBV/HDV co-infection should be suspected when an individual presents with acute hepatitis or liver failure. Antibody to HDV (anti-HDV), HDV RNA or hepatitis D antigen (HDAg) can be checked to establish a diagnosis. HDV RNA is more sensitive especially in the chronic phase when HDAg will complex with anti-HDV and thus may not be detectable. To differentiate super-infection from co-infection, IgM anti-HBc will be positive in the former and negative in the latter [64]. However, some patients with chronic HDV infection can present with relatively mild disturbances in liver enzymes and suppressed HBV viral load [65]. Therefore, we advocate for HDV screening with anti-HDV antibody in all subjects with seropositivity for HBsAg.

#### Risk Determinants and Management of HDV Infection

The risk factors and recommendation measures to prevent spread of HDV transmission among elite athletes mirror those of HBV.

Although significant advances have been made for treatment of chronic hepatitis C virus in the past decade, HDV remains a major challenge. Even though HBV is vital for HDV transmission and disease pathogenesis, suppression of HBV replication by NUCs has a minimal effect on HDV because of the autonomous manner of HDV replication. Moreover, the rate of HBsAg suppression with NUCs is very slow [66], partially due to the expression of HBsAg from HBV integrated DNA, meaning NUCs are ineffective in controlling HDV.

PEG-IFN based therapy has been the mainstay of treatment for HDV. However, response rates are poor, ranging from 25 to 40%, (defined as off-therapy virological control after 1 year of therapy) [67–69]. Moreover, in some subjects, the drug is poorly tolerated, with adverse effects including fatigue, myalgia, influenza-like syndromes, psychiatric complaints and cytopenia [69, 70]. In addition, PEG-IFN is contraindicated in patients with decompensated cirrhosis. However, long-term follow-up studies on the clinical outcome or survival showed improvement and reversal of advanced liver fibrosis after 20 years from initial treatment with PEG-IFN [71]. Conversely, late relapse (4.5 years follow-up) with reappearance of HDV RNA in the blood was observed in 58% of subjects with initial off-therapy viral suppression [72]. So far there are limited data on the long-term outcome of these late relapse patients, however, prolonged suppression of HDV RNA was associated with favorable outcomes even with persistent HBsAg seropositivity [71, 73].

Bulevirtide (formerly Myrcludex-B) is a lipomyristolated peptide that competes with natural HBsAg for the entry receptor sodium taurocholate co-transporting polypeptide on hepatocytes, thereby inhibiting viral entry. Conditional approval was obtained for bulevirtide for medical use as an orphan drug for chronic HBV/HDV coinfection in the European Union in July 2020 and FDA approval was granted in June 2022. In the clinical trials, 24 weeks of various doses of bulevirtide in patients with hepatitis B/D co-infection led to a 2-log decline or undetectable HDV RNA at week 24 in up to 77% of patients receiving 10 mg of bulevirtide compared to only 3% of those receiving NUC. Moreover, 48 weeks of bulevirtide plus PEG-IFN was more effective in achieving undetectable HDV RNA at week 72 (i.e., 24 weeks off-therapy) compared to PEG-IFN monotherapy [74]. The safety and efficacy of bulevirtide have also been demonstrated in patients with compensated cirrhosis [75]. Bulevirtide needs to be administered subcutaneously, but in general is well tolerated even with asymptomatic increases in serum bile salts. Similar to HBV treatment, bulevirtide is not on the Prohibited List of substances according to WADA [29]. Other novel treatment options being evaluated for HDV have been reviewed elsewhere [76].

Overall, HDV causes accelerated liver disease in people with HBV, and treatment options are available aiming to control the disease but responses vary. The prevention of HDV relies heavily on the measures to halt HBV transmission as detailed in the previous section and underlines the importance of HBV vaccination.

### Hepatitis C Virus

#### Epidemiology and Clinical Implications

In the same report published by the WHO, it was estimated that 58 million (0.75% of the global population) were living with HCV in the world. Like HBV, HCV infection is unevenly distributed across the world. The European continent (12 million; 1.3%) and Eastern Mediterranean region (12 million; 1.6%) are the most affected, followed by the Western Pacific (10 million; 0.5%) South-east Asia (10 million; 0.5%), Africa (9 million; 0.8%) and the Americas (5 million; 0.5%) [9].

HCV is an RNA virus and can lead to chronic hepatitis C infection in humans (Table 1). Like HBV, it is transmitted by the percutaneous route and can cause significant liver disease. It is estimated that around 30% of HCV carriers will have progressive liver disease leading to cirrhosis [77]. In the past, HCV infection was classified as acute or chronic (i.e., > 6 months). The term ‘recently acquired HCV’ has replaced ‘acute HCV’ due to difficulty in establishing the exact timing of infection in real life and the potential implications in terms of treatment indication [78]. When an individual is exposed to HCV, the majority will be asymptomatic. However, some patients will present with acute icteric illness, or extra-hepatic manifestations including cryoglobulinaemic vasculitis, lymphoma, cardiovascular disease and insulin resistance [79]. The majority cannot clear the virus from the body, leading to chronic infection in approximately 75% of cases [80]. In 2015, it was estimated that 71 million people globally were living with HCV and 0.4 million deaths were recorded due to HCV infection [2].

#### Diagnosis

Antibody to HCV (anti-HCV Ab) detection in the blood is considered the first-line test and when positive, should be followed by reflex HCV RNA or HCV core antigen assay. Anti-HCV is not a neutralizing antibody and does not protect against re-infection, but it is a marker of exposure to HCV. Therefore, anti-HCV positivity is expected even if an individual has cleared the virus either spontaneously or by treatment. HCV RNA or core antigen testing can confirm active viral replication and thus the diagnosis of HCV is made. By convention, persistence of HCV RNA or core antigen in the blood for > 6 months is defined as chronic HCV infection. Rapid diagnostic tests for either anti-HCV or HCV RNA can aid in improving case identification and access to care [78].

#### Determinants for Risk of HCV Transmission

Although there are virtually no data in the literature regarding HCV transmission or outbreaks among elite athletes, evidence of heightened risk was demonstrated in a study among former female soccer and basketball players in Brazil, where the prevalence of HCV was 7.2% and was related to injectable vitamin complexes or stimulants [81]. The risk of HCV transmission is related to the engagement in at-risk activities or risk exposures, such as injection/intranasal drug use, men who have sex with men and people living with HIV [82]. Outside of these factors, the risk of passing on or acquiring HCV from another athlete is dependent on the level of contact for a particular sport (Table 3).

#### Prevention of HCV Transmission

As there are no vaccines against HCV, avoidance of risk factors, early identification of HCV-infected individuals and the use of finite duration highly-effective direct acting antiviral (DAA) treatment forms the cornerstone of prevention of HCV transmission in elite athletes. The suggested preventive measures are essentially the same as for HBV (see Fig. 3), except that there are no available vaccines against HCV. Among individuals participating in full contact/ collision sports or at high-risk of acquiring hepatitis C virus, screening for HCV infection should be performed regularly.

#### Management of HCV-Infected Elite Athletes

Following the diagnosis of HCV infection, the individual should be referred for specialist evaluation and treatment. In the past, genotype subtyping for HCV was mandatory due to the implications for treatment efficacy. Within less than 3 decades from the discovery of HCV in 1989, remarkable improvement in the understanding of the viral structure and replication cycle led to major advancement in therapeutic strategies. Traditional treatment with PEG-IFN with or without ribavirin is no longer standard-of-care due to the numerous side effects, limited efficacy and the need for subcutaneous injections. The revolutionary all-oral interferon-free DAAs became the new paradigm of HCV treatment, with proven high efficacy and good safety profiles. Several pan-genotypic DAA regimens are now available globally. A full-course of DAA comprises 8 to 12 weeks of therapy and is associated with minimal side effects. DAAs are not on the Prohibited List of substances according to WADA [29]. Unlike HBV, virus clearance can be achieved for HCV and is defined as achieving sustained virological response (SVR), which refers to undetectable serum HCV RNA at 12 to 24 weeks from end of therapy. SVR is durable [83, 84] and can reduce both liver-related complications [85] and extra-hepatic manifestations [86]. Current DAA regimens are associated with more than 90–95% SVR rate for non-cirrhotic treatment-naïve patients [87].

Pre-therapeutic assessment mandates checking liver fibrosis status and liver function. Although decompensated cirrhosis is rare among elite athletes due to their relative young age, the treating physician should exclude cirrhosis to avoid drug toxicity, select the appropriate treatment regime and arrange long-term surveillance for liver-related complications [88–91]. Evaluation for co-infection with other blood-borne viruses should be performed. HIV co-infection is associated with faster disease progression in HCV, and the drugs used for treating HIV and HCV need to be carefully selected because of potential drug–drug interaction. For HBV co-infection, there is risk of HBV reactivation following DAA therapy [92]. NUC for HBV is recommended during DAA therapy for HBsAg-positive individuals until at least 12 weeks post-DAA therapy [78].

Regarding treatment criteria, DAA is recommended for all HCV-positive individuals regardless of liver function or fibrosis status, unless life expectancy is limited because of non-liver-related co-morbidities. Priority for urgent treatment should be given to those with advanced liver disease, extra-hepatic manifestations, history of transplantation, or those at risk of transmitting HCV to others [78]. Following SVR, unless there is pre-existing advanced liver fibrosis, the individual is considered to be cured and no long-term follow-up is required. However, it needs to be emphasized that re-infection should be prevented as anti-HCV is a non-neutralizing antibody. If an individual is repetitively exposed to risk factors, it is recommended that a HCV RNA test should be repeated to detect re-infection. The efficacy of DAA is in general preserved in cases of reinfection as shown by real-world cohort studies [93]. An elite athlete participating in full contact/ collision sports should be allowed to resume training/competition after SVR is achieved.

Overall, HCV is a highly curable disease. Targeted screening and linkage to care in high-risk groups among elite athletes is a reasonable strategy.

## Conclusion

Viral hepatitis type B, C and D can cause significant liver-related morbidity. Transmission of these hepatotropic viruses, in particular HBV, has been documented among elite athletes. Strategies to prevent transmission of HBV, HCV and HDV should adopt the risk-stratification approach taking into consideration the level of contact, known at-risk conditions and epidemiology. Mandatory testing for HBsAg should be implemented for all collision/ contact sports. HBV vaccination should be advocated for all elite athletes and highly viremic HBV-infected individuals should be restricted from playing until the target viral load threshold is reached with antiviral therapy especially for high-risk players. HCV is now curable  with a ≤ 3-month course of antiviral therapy and the best approach is to screen for HCV infection among at-risk individuals. Finally, awareness of HDV should be heightened among people with HBV and any individual found to be HBsAg-positive should have a reflex HDV antibody test. Based on the current evidence, these recommendations should serve as a guide for all athletes participating in collision/ contact sports to minimize the risk of viral hepatitis transmission, and for those infected individuals to be able to participate and compete in sport by ensuring an appropriate standard of specialist care.

## Data Availability

Not applicable.

## Abbreviations

HBV: Hepatitis B virus
HCV: Hepatitis C virus
HDV: Hepatitis D virus
HCC: Hepatocellular carcinoma
WHO: The World Health Organization
CHB: Chronic hepatitis B
HBsAg: Hepatitis B surface antigen
HBeAg: Hepatitis B e antigen
HBV DNA: HBV deoxy-ribonucleic acid
ALT: Alanine aminotransferase
anti-HBc: Antibody to hepatitis B core antigen
cccDNA: Covalently closed circular DNA
copies/mL: Copies per millilitres
HIV: Human immunodeficiency virus
HCP: Health care professionals
EPP: Exposure-prone procedures
UKAP: The United Kingdom Advisory Panel
CDC: The Centers for Disease Control and Prevention
PEG-IFN: Pegylated interferon
NUCs: Nucleos(t)ide analogues
ACIP: Advisory Committee on Immunization Practices
anti-HBs: Antibody to HBsAg
IU/L: International units per litre
mIU/mL: Milli-international units per milli-litre
anti-HCV Ab: Antibody to HCV
DAA: Direct acting antiviral
SVR: Sustained virological response
GLE/PIB: Glecaprevir/pibrentasvir
SOL/VEL/VOX: Sofosbuvir/velpatasvir/voxilaprevir
anti-HDV: Antibody to HDV
HDAg: Hepatitis D antigen
